# Evaluation of Rapidly Disintegrating Vaginal Tablets of Tenofovir, Emtricitabine and Their Combination for HIV-1 Prevention

**DOI:** 10.3390/pharmaceutics6040616

**Published:** 2014-12-08

**Authors:** Meredith R. Clark, M. Melissa Peet, Sarah Davis, Gustavo F. Doncel, David R. Friend

**Affiliations:** 1 CONRAD, Department of Obstetrics and Gynecology, Eastern Virginia Medical School, Arlington, VA 22209, USA; E-Mails: MClark@conrad.org (M.R.C.); doncelGf@evms.edu (G.F.D.); 2 MPI Research, Mattawan, MI 49071, USA; E-Mails: Missy.Peet@mpiresearch.com (M.M.P.); Sarah.davis@mpiresearch.com (S.D.)

**Keywords:** tenofovir, emtricitabine, microbicide, vaginal tablets, pharmacokinetics

## Abstract

Vaginal tablets are being developed as an alternative to gels as an inexpensive, discreet dosage form for the administration of microbicides. This work describes the pharmacokinetic (PK) evaluation of rapidly disintegrating vaginal tablets containing tenofovir (TFV, 10 mg), emtricitabine (FTC, 10 mg), and the combination of TFV and FTC (10 mg each) under *in vitro* and *in vivo* conditions, and in direct comparison to the clinical TFV 1% gel, a microbicide product in Phase III clinical testing. The PK of TFV and FTC from tablets were also evaluated in female rabbits following intravaginal administration. Direct comparison of a single dose of TFV tablets (intact or predissolved at 10 mg/mL) and TFV 1% gel showed no differences in the vaginal PK of TFV between groups; however systemic bioavailability of TFV was significantly higher from the gel. When rabbits were dosed either once or daily for seven days with intact tablets of TFV, FTC, or the combination of TFV/FTC, vaginal and systemic concentrations of TFV and FTC were unaffected by co-formulation. Moreover, plasma PK parameters were similar following a single dose or seven once-daily doses. Tissue concentrations of TFV and FTC in the cranial vagina 4 h after administration ranged between 10^4^ and 10^5^ ng/g. Concentrations of TFV-diphospate (TFV-DP, the active metabolite) were also high (over 10^3^ ng/g or about 3000 to 6000 fmol/mg) in the cranial vagina 4 h after administration and similar to those measured following administration of TFV 1% gel. These data demonstrate that rapidly disintegrating vaginal tablets may be a suitable topical microbicide dosage form providing similar vaginal TFV PK to that of TFV 1% gel. The data also support co-administration of FTC with TFV in a single vaginal tablet to create a combination microbicide in a simple and inexpensive dosage form.

## 1. Introduction

As women in sub-Saharan Africa and other developing world regions continue to be disproportionally affected by the HIV pandemic [1,2], there remains a compelling need to develop acceptable and effective woman-initiated microbicide products. A variety of dosage forms have been proposed and examined as a means to administer vaginal microbicides. The most commonly investigated dosage form has been the vaginal gel, which is relatively easy to develop and simple to administer particularly in a clinical trial setting. In the CAPRISA 004 Phase IIb clinical trial, tenofovir (TFV) 1% gel became the first, and currently only, vaginal microbicide to demonstrate prophylactic efficacy against HIV acquisition [3]. While TFV gel and other vaginal gels are generally accepted by women, gel leakage is a common observation that may reduce not only a women’s desire to use the product but also the amount of drug available at the sites of HIV-1 transmission. Both of these issues have the potential to reduce overall effectiveness of a microbicide product. Poor adherence in particular continues to be a major challenge to the microbicides and oral HIV pre-exposure prophylaxis fields, as it has been linked to the inability to demonstrate efficacy in several clinical studies, including for the once-daily use of TFV gel [4,5].

Another issue to consider for the clinical development of microbicide gels is that they often involve use of a pre-filled, single use plastic applicator. Wide scale access to a gel-based microbicide product could lead to issues around disposal of the non-biodegradable (and biodegradable) applicators. An initial cost effectiveness assessment of TFV 1% gel concluded it may be cost effective in South Africa, though the assumed product costs were probably too low [6].

Alternatives to vaginal gel formulations for prevention of sexual transmission of HIV-1 include intravaginal rings, gelatin ovules, fast-dissolve films, and vaginal tablets/pessaries [7,8,9]. These alternatives have been evaluated in acceptability studies [10,11,12]. Of these options, vaginal tablets represent an acceptable and economical choice for development of alternative vaginal microbicide formulations. Vaginal tablets are available commercially in both the developed and developing world for cervical ripening and treatment of vaginal atrophy (post-menopausal treatment), vulvovaginal candidiasis, and bacterial vaginosis. They have also been studied to some extent as vaginal dosage forms to prevent sexually transmitted infections [13,14,15].

This work was undertaken to develop vaginal tablets as an affordable, acceptable and discreet alternative dosage form to TFV 1% gel and to create a product formulated with two antiretrovirals [TFV and emtricitabine (FTC)]. The tablets are designed to disintegrate rapidly, ideally in less than 30 min, once administered into the vagina. The tablets should also be easy to handle so they can be administered without an applicator.

The studies reported herein were designed to assess the *in vitro* and *in vivo* pharmacokinetics (PK) of the vaginal tablets containing TFV and/or FTC, compared to TFV 1% gel. *In vitro* and *in vivo* PK was performed using organotypic vaginal-ectocervical tissues (EpiVaginal™, MatTek, Ashland, MA, USA) and rabbits, respectively. Parallel safety and PK assessment of these TFV and TFV/FTC tablets was also performed in pigtail macaques [16,17], the results of which are reported elsewhere. In addition, an early prototype of the TFV rapidly disintegrating vaginal tablet was previously evaluated for safety and plasma levels in pigtail macaques [18].

## 2. Materials and Methods

### 2.1. Materials

TFV and FTC drug substances were obtained from Gilead Sciences (Foster City, CA, USA). Tablet excipients (mannitol, microcrystalline cellulose, crospovidone, hydroxyethyl cellulose, and sodium stearyl fumarate) were either United States Pharmacopeia (USP) or National Formulary (NF) grade. TFV-d6, cladribine, and emtricitabine-^13^C^15^N_2_, and (−)-emtricitabine-^13^C^15^N_2_^13^CH_10_FN^15^N_2_O_3_S (internal standards) were obtained from Toronto Research Chemicals, Inc. (Toronto, ON, Canada). TFV–diphosphate (TFV-DP) and TFV-DP, adenine-^13^C_5_ were purchased from Moravek Biochemicals, Inc. (Brea, CA, USA). Weck-Cel^®^ surgical spears were obtained from Medtronic Ophthalmics (Jacksonville, FL, USA). Organotypic human vaginal-ectocervical tissues (EpiVaginal™ tissues; VEC-100-FT) were obtained from MatTek Corporation (Ashland, MA, USA). Dulbucco’s phosphate buffered saline (DPBS) was obtained from Invitrogen (Carlsbad, CA, USA). Gynol II (Advanced Care Products, Ortho Pharmaceutical Corp., Raritan, NJ, USA) was purchased commercially.

### 2.2. Vaginal Tablets and Gel

Rapidly disintegrating tablets were prepared using a standard rotary tablet press following wet granulation. The diameter of the tablets was 8.0 mm (TFV and FTC) or 9.0 mm (TFV/FTC combination) with both faces flat. Tablets were prepared with TFV (10 mg), FTC (10 mg) or TFV/FTC (10 mg/10 mg). The total mass of each tablet was approximately 125 mg. Tablets were analyzed for assay, moisture content, disintegration time following USP 35/NF 30 <701>, hardness, and friability (Table 1). The tablets were prepared by Aptalis Pharmtech (Vandalia, OH, USA). TFV 1% gel (same formulation as used in the CAPRISA 004 study) was prepared by DPT Laboratories, Inc. (San Antonio, TX, USA) or Patheon Pharmaceuticals, Inc. (Cincinnati, OH, USA).

### 2.3. In Vitro Permeability

The tissue permeability of TFV, FTC and TFV/FTC tablets was assessed using the organotypic human vaginal-ectocervical (MatTek, VEC-100 FT) tissues as described previously [19]. Tablets were suspended in either 0.5 or 1.0 mL DPBS (pH 7.40 ± 0.05) by vortexing for 5 s. The tablets were completely disintegrated by this process. Aliqouts (100 µL) of these mixtures were applied to the apical side of the VEC-100-FT tissues. TFV and FTC were measured in the tissues (tissue-associated), rinses, and receptor phases over a 24 h period of exposure tablet suspensions. TFV was quantified using liquid chromatography-tandem mass spectrometry (LC/MS–MS) as previously reported [20]. The method has a lower limit of quantitation of 10 nM (3.05 ng/mL). FTC was measured using an LC/MS–MS method described as follows. Prior to bioanalysis, samples (receptor fluids and cell lysates) were prepared as described for TFV previously [20]. The method was developed and validated according to the FDA Guidance for Industry: Bioanalytical Method Validation, May 2001. Analysis of samples was performed using an Applied Biosystems API 4000 LC/MS–MS (Foster City, CA, USA) operated in the positive mode, Perkin Elmer Series 2000 pumps, and a CTC Analysis autosampler. The column used was a Thermo BioBasic AX, 50 × 2.1 mm, 5 µm (Part No. 73105-052130) with an in-line frit filter (0.5 µm, 0.062 × 0.065 × 0.2485 in., Upchurch Scientific, Oak Harbor, WA, USA). The column was held at ambient room temperature (~22 °C); the injection volume was 10 µL. A gradient method was used with a flow rate of 300 µL/min. Two mobile phase compositions were used: A, acetonitrile: 10 mM ammonium acetate, pH 6.0 (70:30, *v*/*v*) and B, acetonitrile: 1 mM ammonium acetate, pH 10.5 (60:40, *v*/*v*). The gradient method initiated at 80% A/20% B and was changed to 60% A/40% B after 1 min, 50% A/50% B at 1.4 min, and 10% A/90% B at 2.8 min. Under these conditions, the retention time of FTC and the internal standard, cladribine was ~1.5 min. The lower limit of quantitation (LLOQ) of FTC was 10 nM (2.48 ng/mL).

**Table 1:** Physicochemical properties of tenofovir (TFV), emtricitabine (FTC) and TFV/FTC tablets used in EpiVaginal and rabbit studies.

Description	TFV Tablets	FTC Tablets	TFV/FTC Tablets
	White to off-White, Round, Flat Faced Tablet		
Drug Dose	10 mg TFV	10 mg FTC	10 mg TFV/10 mg FTC
Assay (% Label Dose)	97.2%	102.3%	101.9% TFV/100.8% FTC
Total Mass	~125 mg	~125 mg	~125 mg
Diameter	8 mm	8 mm	9 mm
Disintegration Time ^a^	60–75 s	60–75 s	60–75 s
Moisture Content	2.6%	1.7%	2.2%
Hardness (N)	28	97	14
Friability (%)	0.5%	0.1%	0.4%

^a^ Determined using USP 32 <701> method.

### 2.4. Pharmacokinetics in Rabbits

**Figure 1:**
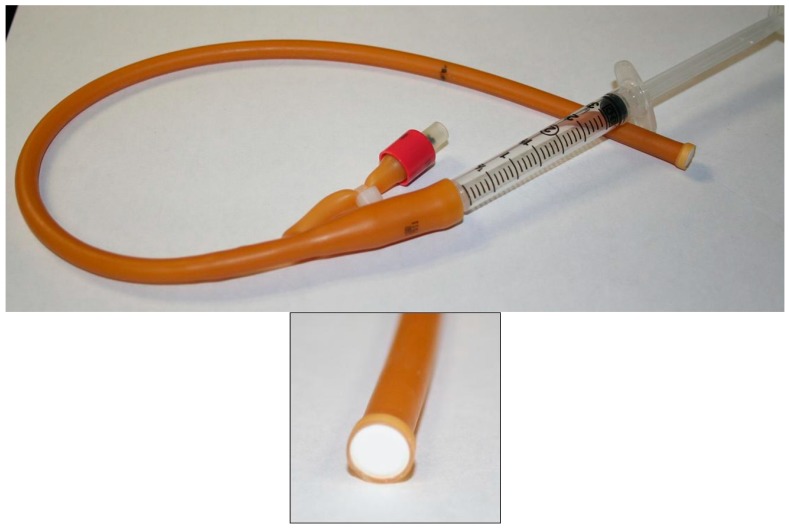
Tablet dosing apparatus; Inset: tablet loaded in tip of device. The apparatus is constructed from a modified Foley catheter (18 or 20 French). After insertion into the abdominal vagina (~8 cm, distance marked by black line on catheter), the tablet is released from the device using gentle air pressure generated by an attached disposable syringe.

The PK assessment of the vaginal tablets in rabbits consisted of two studies under a single protocol (1645-074) and approved by MPI Research’s IACUC on 2 September 2011. The first study involved evaluation of TFV (10 mg) administered as an intact tablet, tablet powder blend suspended in 1.0 mL PBS, or aqueous gel (1.0 mL TFV 1% gel). Groups of five rabbits each were dosed once and sacrificed at various times (0.5, 4, 8, and 24 h post-dose). Plasma, vaginal tissues (cranial and caudal), vaginal fluids, iliac lymph nodes were sampled and analyzed for TFV. Study 2 involved the assessment of PK following administration of one or seven daily doses of intact tablets containing TFV (10 mg), FTC (10 mg), or the combination TFV/FTC (10 mg/10 mg). The same matrices were examined for levels of TFV or FTC. Details of the use of animals are described elsewhere [21]. Intact tablets were administered to the upper (cranial) vagina using a custom-made applicator device as shown in Figure 1. This device consisted of a 3 mL luer lock syringe equipped with a modified Foley catheter (18–20 French, or 6–6.67 mm). The tablet was affixed to the end of the catheter, as shown in Figure 1. Once the tablet-loaded catheter was gently inserted into the vagina approximately 8 cm from the introitus, the tablet was dislodged from the catheter by gently pressing the syringe plunger, providing a short burst of air pressure to push the tablet out of the catheter. This procedure required the animals to be mildly sedated with acepromazine (0.3 to 0.5 mg/kg) administered via intramuscular injection about 5 min prior to dosing. Predissolved tablet powder blends and gel were administered using a catheter as described previously [19,21].

### 2.5. Bioanalysis

Sample preparation and bioanalytical methodology for TFV in plasma, vaginal fluid, and iliac lymph nodes have been described elsewhere [21]. A modified method was used to analyze TFV and TFV-DP in vaginal tissues. Tissues stored frozen at −70 to −80 °C were processed in a small blade homogenizer with dry ice to prepare a finely ground tissue sample. The samples were placed overnight at −20 °C to allow the dry ice to sublime. The ground tissue was then diluted with acetonitrile (ACN):H_2_O 10-fold (100 mg tissue: 1 mL of solvent). The samples were then sonicated for 15 min in an ice water-bath. The samples were then centrifuged at 4000 rpm at 4 °C for 10 min. The samples were then allowed to thaw to room temperature. Each sample was then vortexed and centrifuged prior to aliqouting. Next, 200 µL of standards, quality control standards, sample, or blanks were added to a 96-well extraction plate. To these wells (except blanks) were added the working internal standards (TFV-d6 or TFV-DP, adenine ^13^C_5_) (50 µL of a 100 ng/mL solution in water). The plate was then capped and vortexed for ~30 s. The plate was then centrifuged at ~4000 rpm for 5 min at 4 °C.

Bioanalytical analysis of the samples was performed by LC/MS–MS using an Agilent 1100 series HPLC system coupled with an Applied Biosystems/MDS Sciex API 5000 system (Thermo Fisher Scientific, Inc., Waltham, MA, USA). MS/MS data were collected in the positive polarity mode. The analytical column used was a BioBasic AX column (Thermo Fisher Scientific, Inc., Waltham, MA, USA) 50 × 3.0 mm, 5 µm. A gradient mobile phase was used were Phase A was ACN/10 mM ammonium acetate in water (pH 6.0) (30:70) and Phase B was ACN/1 mM ammonium acetate in water (pH 10.5) (30:70). The initial mobile phase from 0 to 1.0 min was A/B (95:5) which was changed to 50:50 from 1.0 to 2.0 min. From thereafter the mobile consisted of only Phase B. The flow rate was 400 µL/min and the column was at ambient room temperature. The autosampler was held at 5 °C. Under these conditions, the retention time of TFV was 4.91 min and TFV-DP was 5.80 min. The LLOQ for TFV was 20 ng/g and that of TFV-DP was 100 ng/g.

Bioanalysis of FTC in rabbit plasma, vaginal tissues, and vaginal fluids were all validated according to the FDA Guidance for Industry: Bioanalytical Method Validation, May 2001.

The measurement of FTC in plasma in K_3_EDTA containing tubes stored at −50 to −90 °C was accomplished as follows. The samples were thawed to room temperature and vortexed followed by aliqouting 50 µL into a 96-well extraction plate. The internal standard, (−)-emtricitabine-^13^C^15^N_2_^13^CH_10_FN^15^N_2_O_3_S (500 ng/mL), was then added to each well (50 µL). Then 25 µL of trifluoroacetic acid was added to all plate wells containing plasma. The plate was then capped and gently vortexed for approximately 20 s. The plate was then left to stand for 15 min at ambient room temperature. Water (400 µL) was added to each well followed by mixing using gentle vortex for approximately 20 s. The plate was then centrifuged at 20 °C at 4500 rpm for 15 min. Next concentrated NH_4_OH (20 µL) was added to a clean 96-autosampler well plate. The supernatant from the samples (300 µL) was then added on top of the 20 µL of the NH_4_OH. The plate was then gently mixed by vortexing for approximately 20 s. The plate was centrifuged at 20 °C at 4500 rpm for 15 min. The samples were then analyzed by LC/MS–MS uing an Agilent 1100 HPLC system and an Applied Biosystems/MDS SCIEX API 5000 mass spectrometer operated in the positive polarity mode. The column used was a Phenomenex Synergi Polar-RP 2.0 × 75 mm, 4 µm, 80 Å. The mobile phase (isocratic) consisted of water/acetonitrile/acetic acid/ammonium hydroxide (930:70:5:1, *v*/*v*/*v*/*v*). The flow rate was 200 µL/min; column temperature was ambient temperature as was the autosampler. The injection volume was 10 µL. Under these conditions, the LLOQ was 1.0 ng/mL and an upper limit of quantitation of 1000 ng/mL. The retention time of FTC was 3.7 min.

The bioanalysis of FTC in vaginal fluids was the same as that described previously for TFV in vaginal fluids [21]. The LLOQ of FTC was 5.0 ng/spear and the ULOQ was 500 ng/spear. The retention time of FTC was 3.3 min.

The bioanalysis of FTC in tissues was performed as follows. Weighed tissues (snap frozen and stored at −70 to −80 °C) were processed in small blade homogenizer with dry ice to make a finely ground sample. The sample was placed in a freezer (approximately −20 °C) overnight to allow the dry ice to sublime. The ground tissue was then diluted with ACN:water (50:50) 10 fold (maintaining a ratio of 100 mg tissue: 1 mL of the solvent). The mixture was then sonicated (15 min) in an ice water bath. This step was followed by centrifugation at 4000 rpm and 4 °C for 10 min. Samples (100 µL) were then added to a 96-well extraction plate. Then 400 µL working internal standard (20 ng/mL of 1% acetic acid in water) was added to all the wells. This mixture was then transferred to pre-conditioned (400 µL methanol followed by 400 µL of water drawn through with minimal vacuum) solid phase extraction (SPE) plates (Oasis MCX 96-well plate, 30 µm, 10 mg) using a multichannel pipette. Minimal vacuum was applied to pull the samples through SPE. Water (200 µL) was then placed in each well and allowed to flow through with minimal vacuum. The plate was then dried using high vacuum for 2 min. A new 96-well collection plate was used to collect the eluate (300 µL of 1% NH_4_OH in methanol) by again applying minimal pressure until all the wells appeared dry. The 96-well collection plate was then dried using an airflow of ~40 L/min at ~45 °C until completed evaporated. Then, water (100 µL) was added to each well, the plate capped, and vortexed for approximately 30 s. Samples were then analyzed by LC/MS–MS using the same equipment described for plasma analysis and operated in the positive polarity mode. The mobile phase (A) was water/acetic acid/ammonium hydroxide (925:5:1, *v*/*v*/*v*) while mobile phase B was 100% methanol. The flow rate was 0.4 mL/min starting with A at 95% and B at 5%. Between 1.5 and 2.0 min the gradient was changed to 10% A and 90% B. Between 3.5 and 4.0 min the gradient was returned to the initial balance of 95% A and 5% B. The column was held at 40 °C while the autosampler was held at 5 °C. The injection volume was 5.0 µL. Under these conditions, the retention time of FTC was 2.3 min. The LLOQ was 20 ng/mL and the ULOQ was 1000 ng/mL.

### 2.6. Statistics

Statistical comparisons of *in vitro* PK and safety and *in vivo* PK data were performed using ANOVA with Bonferroni multiple comparison test (Origin Ver. 8.0, OriginLab Corporation, Northhampton, MA, USA ).

## 3. Results

### 3.1. Physicochemical Characterization

Certain physicochemical properties of the vaginal tablet formulations are summarized in Table 1. The vaginal tablets were found to disintegrate quickly under standard USP testing conditions. Also, the three formulations were stable based on assay and dissolution time. Note that both drugs are quite soluble at pHs above 5.0 (>100 mg/mL).

### 3.2. In Vitro Permeability

**Figure 2:**
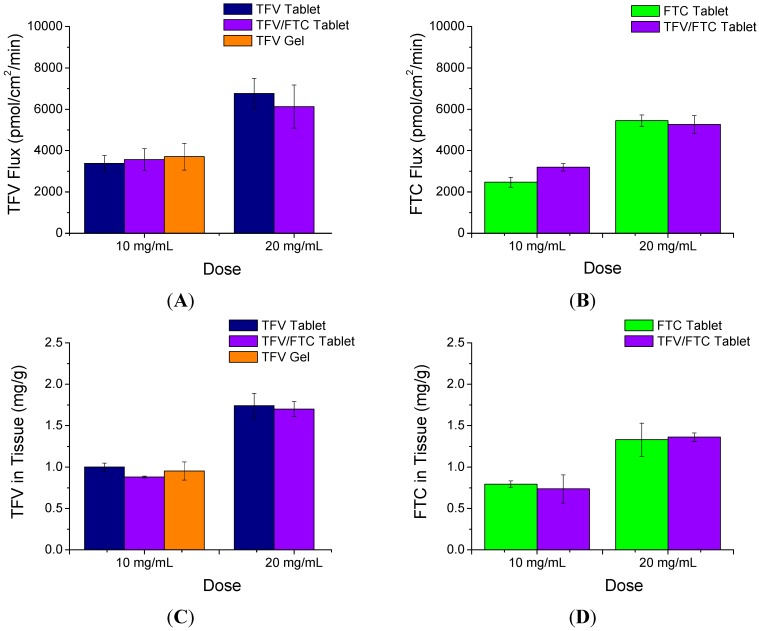
*In vitro* tissue permeation of TFV and FTC following 24 h treatment of EpiVaginal tissues with tablets pre-dissolved at two concentrations compared to TFV 1% gel. TFV (**A**); and FTC (**B**); flux across the tissue and tissue associated concentrations of TFV (**C**); and FTC (**D**) are shown. Tissues were rinsed twice prior to collection of tissues to remove residual drug product on tissue surface. Mean ± SD (*n* = 3).

The rapidly disintegrating tablets were evaluated using organotypic human vaginal ecotocervical tissues. The permeability of TFV and FTC was assessed along with the amount of drug associated with the tissues following 24 h of exposure to tablets suspended in either 0.5 or 1.0 mL BPBS (Figure 2). There were no statistically significant differences between TFV flux from suspensions containing TFV with and without FTC at the same volume (*p* < 0.05). Likewise, the tissue-associated TFV concentrations were similar from suspensions containing TFV alone or in combination with FTC. Moreover, no difference was observed in either the flux or tissue levels of TFV when comparing TFV tablets and TFV gel when dosed at the same concentration (10 mg/mL). When comparing FTC and TFV/FTC combination tablets, there were also no statistically significant differences in FTC flux or amount of FTC associated with tissues from the same volume of suspension. Both flux and tissue associated concentrations for both TFV and FTC increased proportionally with their dosed concentrations in the tablet suspensions (flux increased 1.6- to 2.2-fold and tissue concentrations increased 1.7- to 1.9-fold when dose concentrations were increased from 10 to 20 mg/mL).

### 3.3. In Vivo Pharmacokinetics—Study 1

**Figure 3:**
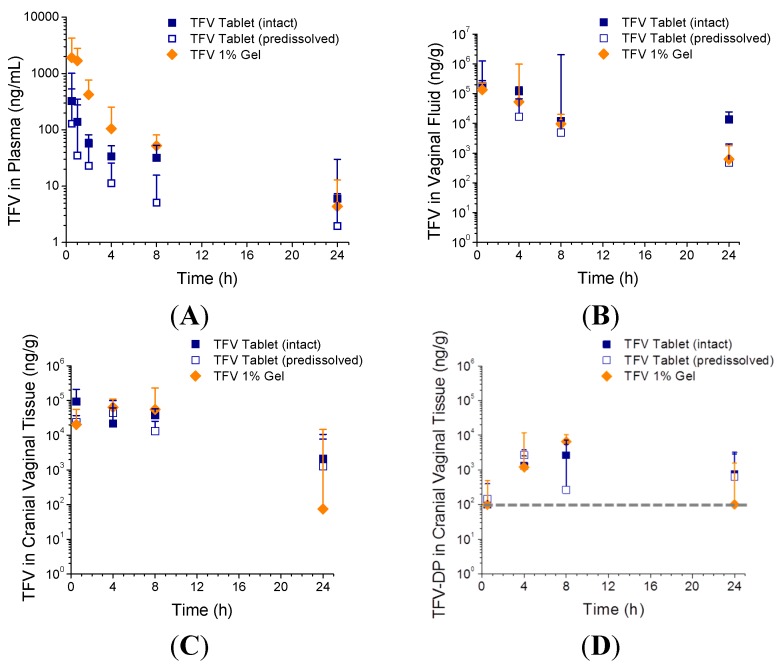
TFV concentrations in plasma (**A**); vaginal fluid (**B**); and cranial vaginal tissue (**C**); and TFV-diphospate (TFV-DP) concentrations in cranial vaginal tissue (**D**) following vaginal administration of TFV tablets, intact or pre-dissolved, and TFV 1% gel (Study 1). Data are medians + SD (*n* = 5). Dashed line denotes the lower limit of quantitation (100 ng/g).

The plasma and vaginal fluid concentrations from Study 1 (comparison of results from vaginal administration of TFV 1% gel, intact TFV tablets, or predissolved TFV tablets) are shown in Figure 3A,B. The mean plasma PK parameters are summarized in Table 2. The difference in *T*_max_ between the three formulations was statistically insignificant (*p* = 0.56). *C*_max_ for the gel was 3- to 8-fold higher than the tablet formulations, however this difference was not statistically significant (*p* = 0.09) due to dispersion of the data. The difference in plasma AUC_0–24 h_ between groups was statistically significant, with AUC_0–24 h_ for the gel being 4-fold higher than that from either the intact or predissolved tablets (*p* = 0.05 and 0.04, respectively). There was no sign of tablet or residue leakage over the course of the study.

**Table 2:** TFV plasma pharmacokinetic (PK) parameters following a single dose intravaginal administration of 10 mg TFV tablets, intact *versus* predissolved (10 mg/mL), and 1 mL TFV 1% gel (Study 1).

Formulation	*T*_max_ (h)	*C*_max_ (ng/mL)	AUC_0–24 h_ (ng·h/mL)
TFV Tablet (intact)	0.70 ± 0.27 ^a^	297 ± 199	858 ± 342
TFV Tablet (pre-dissolved)	1.20 ± 1.57	694 ± 879	778 ± 802
TFV 1% Gel	0.60 ± 0.22	2352 ± 2300	3615 ± 2558

^a^ Data are mean ± SD (*n* = 5).

Median TFV concentrations in vaginal fluid (collected from the caudal vagina) following vaginal administration of the intact TFV tablets peaked at approximately 10^5^ ng/g at 30 min post-dose, and decreased to approximately 10^4^ ng/g by 24 h (Figure 3C). Vaginal fluid concentrations were similar between TFV tablets and TFV gel at all timepoints, with the exception of the intact TFV tablet providing over 20-fold higher fluid levels than either the predissolved tablets or TFV gel at 24 h post dose (*p* = 0.04, Figure 3B).

The TFV and TFV-DP tissue concentrations in the cranial vagina at the various time points evaluated following administration of the three TFV formulations are shown in Figure 3C,D. Similar data from the caudal vagina are located in the supplemental section. Median TFV levels in cranial vaginal tissue were on the order of 10^4^–10^5^ ng/g through the first 8 h post-dose, and dropped to 2 × 10^3^ ng/g by 24 h for the intact TFV tablet group. Caudal TFV tissue concentrations were only slightly lower, with TFV levels on the order of 10^4^ ng/g sustained through 8 h post-dose but dropping to 2 × 10^2^ ng/g at 24 h for the same group. There were no differences statistically at any time point in either the cranial or caudal TFV tissue concentrations between the tablet and gel formulations, however median TFV values tended to be higher (17- to 28-fold) in the tablet groups compared to the gel group at 24 h post dose. Median TFV-DP levels in cranial vaginal tissue varied from below the limit of quantitation (BLQ < 100 ng/g, or <224 fmol/mg) to approximately 7 × 10^3^ ng/g (1.5 × 10^4^ fmol/mg). There were no differences in the TFV-DP tissue levels in the cranial vagina, however more samples were BLQ for the gel group at 24 h post-dose compared to the tablet groups (four of five samples were BLQ for the gel group, compared to one or two samples out of five were BLQ for the tablet groups). In the caudal vagina, more samples were near or BLQ across all groups, making comparisons difficult.

The median concentration of TFV in the iliac lymph nodes was sustained at approximately 10^2^ ng/g for most groups through the 24 time period following dosing (see Supplementary Information). There were no differences statistically between the three formulations at any time point tested.

### 3.4. In Vivo Pharmacokinetics—Study 2

**Figure 4:**
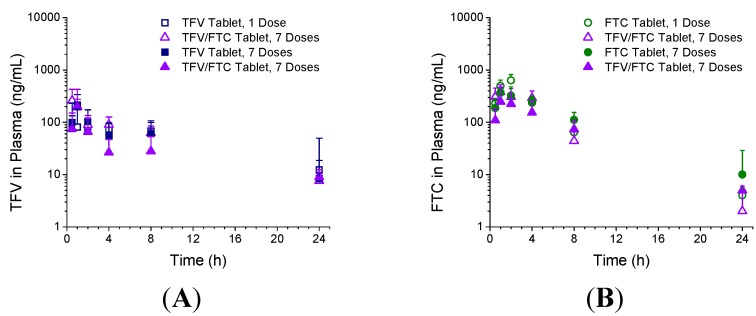
TFV (**A**); and FTC (**B**) plasma concentrations following vaginal administration of single entity (TFV or FTC) or combination TFV/FTC tablets (Study 2) following administration of a single tablet (1 Dose) or seven once-daily doses (7 Doses). Data are median + SD (*n* = 6).

The second rabbit study evaluated the PK of TFV and FTC from intact tablets, formulated alone or in combination, following a single or seven once-daily intravaginal doses. TFV plasma concentrations over time following vaginal administration of TFV and TFV/FTC tablets are shown in Figure 4A; FTC plasma concentrations over time following vaginal administration of FTC and TFV/FTC tablets are shown in Figure 4B. Plasma PK parameters are summarized in Table 3 (TFV) and Table 4 (FTC). There were no differences statistically in any parameters when the drugs were administered alone or in combination, or once *versus* daily.

**Table 3:** TFV plasma PK parameters following intravaginal administration of intact TFV tablets (10 mg), or TFV/FTC tablets (10 mg/10 mg), after a single dose and seven once-daily doses (Study 2).

Formulation	*T*_max_ (h)	*C*_max_ (ng/mL)	AUC_0–24 h_ (ng·h/mL)
**Single Dose**			
TFV Tablet	5.83 ± 9.36 ^a^	139 ± 91.9	1458 ± 555
TFV/FTC Tablet	0.667 ± 0.258	249 ± 150	1304 ± 416
**Seven Doses**			
TFV Tablet	2.17 ± 2.86	242 ± 123	1534 ± 489
TFV/FTC Tablet	0.917 ± 0.204	235 ± 231	849 ± 553

^a^ Data are mean ± SD (*n* = 6).

**Table 4:** FTC plasma PK parameters following intravaginal administration of FTC tablets (10 mg) or TFV/FTC tablets (10 mg/10 mg) after a single dose and seven once-daily doses.

Formulation	*T*_max_ (h)	*C*_max_ (ng/mL)	AUC_0–24 h_ (ng·h/mL)
**Single Dose**			
FTC Tablet	2.17 ± 0.983 ^a^	567 ± 185	2992 ± 1027
TFV/FTC Tablet	1.50 ± 1.22	407 ± 142	2361 ± 692
**Seven Doses**			
FTC Tablet	1.33 ± 0.516	403 ± 123	2822 ± 170
TFV/FTC Tablet	1.0 ± 0.0	279 ± 223	1854 ± 971

^a^ Data are mean ± SD (*n* = 6).

**Figure 5:**
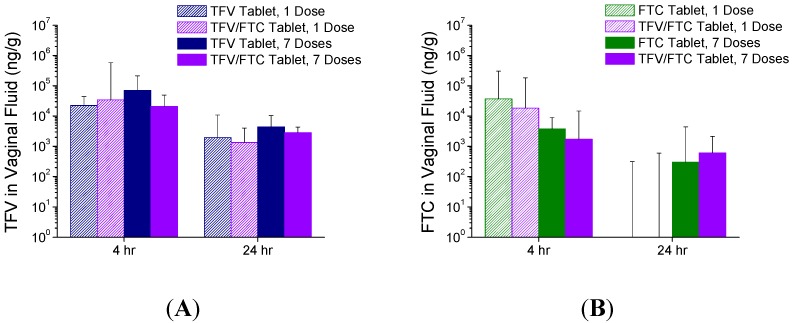
TFV (**A**); and FTC (**B**) vaginal fluid concentrations following vaginal administration of single entity (TFV or FTC) or combination TFV/FTC tablets (Study 2). Data are median + SD (*n* = 6).

TFV vaginal fluid concentrations (again collected from the caudal vagina) following administration of TFV and TFV/FTC tablets are shown in Figure 5A; likewise FTC vaginal fluid concentrations following administration with FTC and TFV/FTC tablets are shown in Figure 5B. At 4 h post-dose, median TFV and FTC levels were typically on the order of 10^3^–10^4^ ng/g, with generally slightly lower levels observed at 24 h. No significant differences were observed for TFV or FTC levels when comparing dosing with either the single entity or combination tablets. Moreover, when comparing single *versus* daily dosing, no differences were observed in the TFV levels at either timepoint. However, FTC levels were observed to be mostly near or BLQ (<5 ng/spear, or approximately 300 ng/g based on an average vaginal fluid swab mass of 15–20 mg) at 24 h following a single dose, whereas half of the 7-day samples were quantifiable and in the 10^3^–10^4^ ng/g range (medians of 300–600 ng/g) at this time point.

**Figure 6:**
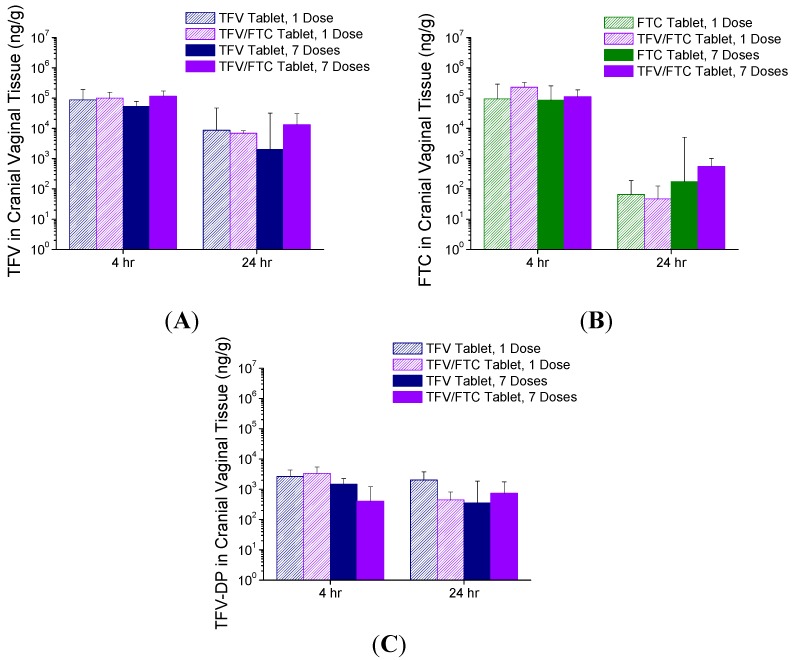
TFV (**A**); FTC (**B**); and TFV-DP (**C**) levels in cranial vaginal tissue concentrations following vaginal administration of single entity (TFV or FTC) or combination TFV/FTC tablets (Study 2). FTC-TP levels were BLQ < 30 ng/g. Data are median + SD (*n* = 6).

Cranial vaginal tissue concentrations of TFV and FTC are shown in Figure 6A,B, respectively. Concentrations of TFV and FTC in caudal vaginal tissues, as well as TFV concentrations in iliac lymph nodes, are located in the Supplementary Information. Median TFV and FTC concentrations in cranial vaginal tissue were both on the order of 10^5^ ng/g at 4 h post-dose. By 24 h post dose, TFV levels dropped approximately one order of magnitude, whereas FTC levels dropped by approximately two orders of magnitude. The concentrations of TFV or FTC in the tissues were insignificantly different at both time points when administered as the single entity tablet or combined with the other drug, or as a single dose compared to after seven daily doses.

The levels of TFV-DP in cranial vaginal tissues are shown in Figure 6C. Similar data from the caudal vagina are found in the Supplementary Information. The median levels were on the order of 10^2^–10^3^ ng/g at both 4 and 24 h following a single dose or seven once-daily doses, with minimal differences observed between single entity and combination tablet groups. The TFV-DP concentrations in the caudal vagina were about an order of magnitude lower compared with the cranial vaginal tissues, and were mostly BLQ at the 24 h timepoint (see Supplementary Information). FTC-TP was also analyzed in vaginal tissues, however most samples were BLQ (<30 ng/g).

## 4. Discussion

This work was aimed at assessing the PK of rapidly disintegrating vaginal tablets for TFV and the combination of TFV and FTC. Both *in vitro* and *in vivo* studies were used, as was direct head-to-head comparison to TFV 1% gel. This work is part of a larger effort to develop an inexpensive and more convenient to use alternative to TFV 1% gel and one that can also deliver FTC (the second drug in the antiretroviral combination Truvada^®^, Gilead, Foster City, CA, USA). The combination of TFV and FTC has been found to synergistically enhance anti-HIV activity while reducing the potential for development of resistance [22,23]. FTC is unstable in water at room temperature or higher; therefore a gel formulation is unsuitable.

The tablet preparations were first evaluated *in vitro* using the organotypic human vaginal ectocervical tissues. The flux and tissue-associated concentrations of TFV and FTC were similar in magnitude yet independent of each other, and were linearly dependent on dose concentration, as expected in terms of permeability. When TFV and TFV/FTC tablets were prepared at the same TFV concentration as TFV 1% gel, both the flux and tissue levels of TFV were comparable across the two dosage forms. Though beyond the scope of this manuscript, the effects of the tablets on the viability and tissue integrity of these organotypic human vaginal ectocervical tissues were also assessed and found to be minimal or similar to TFV gel (data not shown).

*In vivo* PK was assessed in two rabbit studies. In the first study, TFV was administered as a single dose to rabbits in one of three forms: intact tablets, predissolved tablets, and TFV 1% gel. In the second study, intact tablets containing TFV, FTC or the combination of both, were administered as a single dose or seven once-daily doses. The major findings from these two studies are the following: (1) systemic bioavailability of TFV was higher following intravaginal administration of the gel compared to TFV tablets; (2) the plasma concentrations were somewhat higher from intact tablets compared with predissolved tablets perhaps due to the higher concentration gradient in the intact tablets; (3) vaginal PK of TFV was otherwise similar between tablet and gel dosage forms, including those reported previously for TFV 1% gel and reduced-glycerin TFV 1% gel [21], with perhaps the one exception that TFV vaginal fluid and tissue levels appear to be more sustained at 24 h following tablet dosing; (4) TFV and FTC systemic PK from tablets were generally low and independent of formulation (single entity *vs.* combination) and dosing regimen (single *vs.* daily); and (5) TFV and FTC vaginal fluid and tissue concentrations were also independent of formulation and, except for FTC in vaginal fluid, dosing regimen.

Tenofovir 1% gel has been proven to be efficacious in preventing HIV infection in both macaques and humans [24,25]. This protection has been associated with TFV and TFV-DP levels similar to those observed for TFV tablets in the studies described above [24,26]. Given the direct *in vitro* and *in vivo* PK comparisons to TFV 1% gel, these PK results for TFV and FTC are very encouraging. It is unclear whether the differences in rabbit systemic TFV exposure are meaningful in terms of safety/efficacy or if it will be observed in women. It is also unclear whether the trends showing improved vaginal retention of TFV in the rabbit model will also translate to the clinic. However, it should be noted that similar observations were made in a parallel pigtail macaque study comparing TFV gel, TFV tablets and TFV/FTC tablets.

Little is known about the metabolism of TFV to TFV-DP or FTC to FTC-TP in rabbit vaginal tissues and if there are metabolic differences over the length of the vagina. In these rabbit studies, concentrations of TFV-DP in cranial vaginal tissue were measured in excess of 10^3^ ng/g. When expressed as fmol/mg [26], median TFV-DP concentrations in the cranial vagina at 4 h post-dose were 2900, 5900, and 2700 fmol/mg from the tablet, pre-dissolved tablets, and gel, respectively. Median concentrations in the caudal vaginal tissue were generally lower than those measured in the cranial vagina. For FTC-TP analysis, most vaginal tissue samples were observed to be BLQ (<30 ng/g); it remains unclear whether this is a function of differential rabbit metabolism or an artifact of metabolite instability during sample collection and handling.

## 5. Conclusions

The vaginal tablets performed in most regards similarly to TFV 1% gel when evaluated for PK in the rabbit model. Moreover, coformulation of TFV and FTC did not affect the PK of either drug. A Phase I clinical safety/PK trial of these tablet formulations is currently ongoing (ClinicalTrials.gov #NC01694407). In this first-in-women study, the individual dose of TFV and FTC is 40 mg each. The tablets are also somewhat larger (12 mm diameter; 500 mg total tablet weight) than those tested in the studies reported herein. These tablets represent a promising alternative to TFV 1% gel should they prove safe and effective in women.

## Supplementary Files

Supplementary File 1
