# Supplementary material for: Evaluation of Rapidly Disintegrating Vaginal Tablets of Tenofovir, Emtricitabine and Their Combination for HIV-1 Prevention
Source: Pharmaceutics. 2014 Dec 8;6(4):616–31. doi: 10.3390/pharmaceutics6040616 (PMC4279136; doi:10.3390/pharmaceutics6040616)
Supplement: Supplementary File 1 [file pharmaceutics-06-00616-s001.pdf]

Supplementary Information

Figure S1. TFV (top) and TFV-DP (bottom) concentrations in the caudal rabbit vaginal tissue following a single dose of TFV tablet, intact or predissolved, or TFV 1% gel (10 mg TFV each). Data are median + SD ($n = 5$). Dashed line denotes the lower limit of quantitation (100 ng/g).

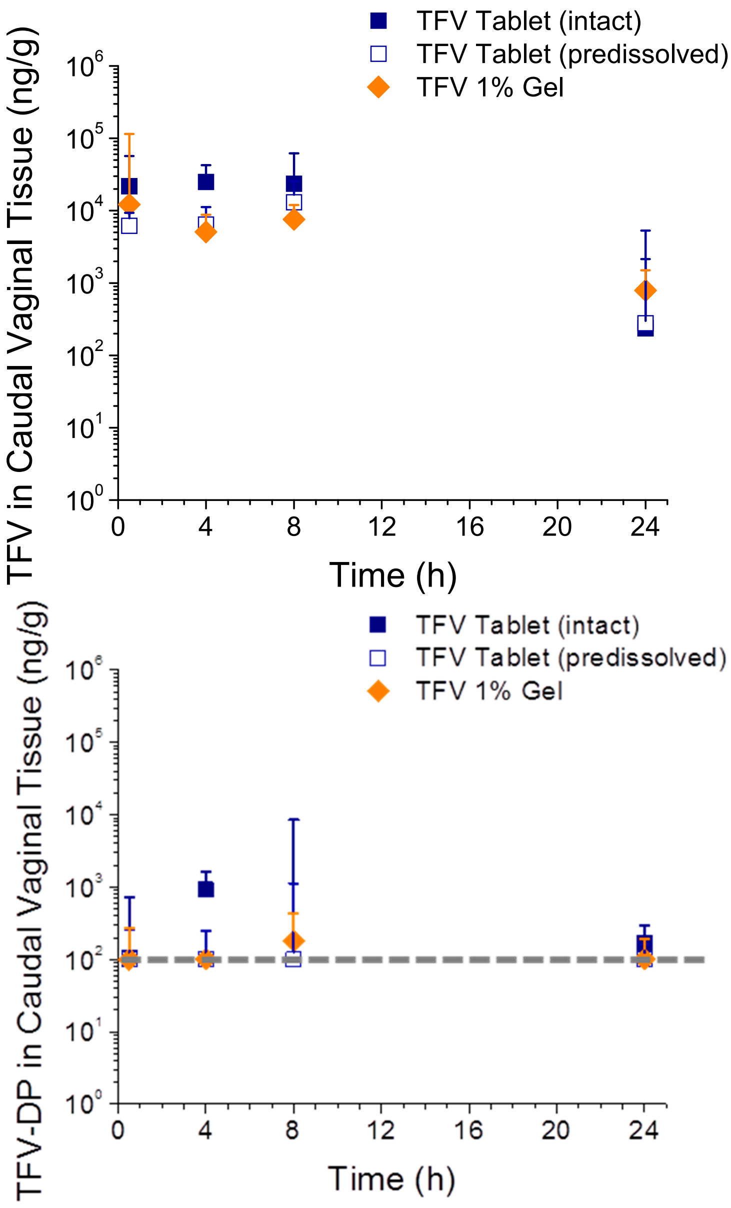

Figure S2. TFV iliac lymph node concentrations vagina following a single dose of TFV tablet, intact or predissolved, or TFV 1% gel (10 mg TFV each). Data are median + SD (n = 5). Dashed line denotes the lower limit of quantitation (20 ng/g).

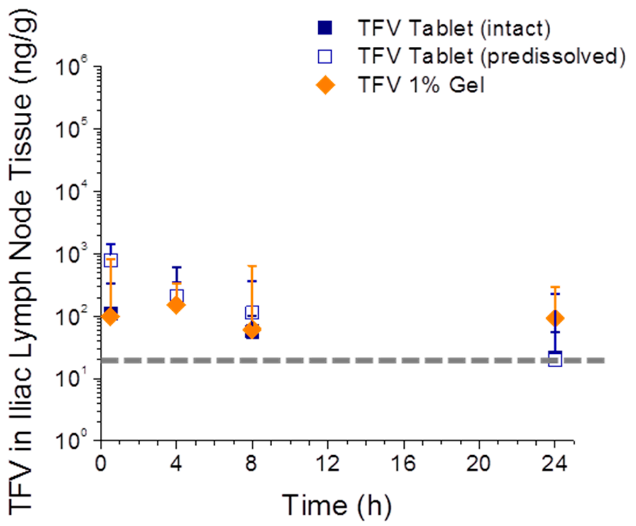

Figure S3. TFV (top) and TFV-DP (bottom) concentrations in caudal vaginal tissues at 4 and 24 h following a single or 7 daily doses of intact TFV or TFV/FTC tablets. Data are median + SD (n = 6). Dashed line denotes the lower limit of quantitation (100 ng/g).

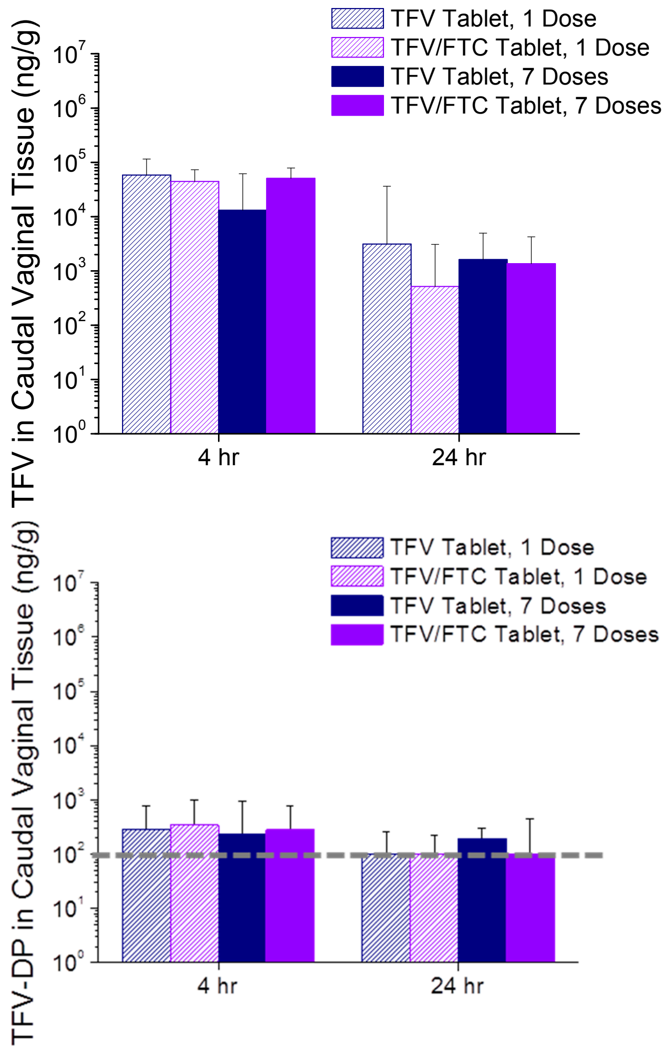

Figure S4. FTC concentrations in caudal vaginal tissues at 4 and 24 h following a single or 7 daily doses of intact FTC or TFV/FTC tablets. Data are median + SD (n = 6).

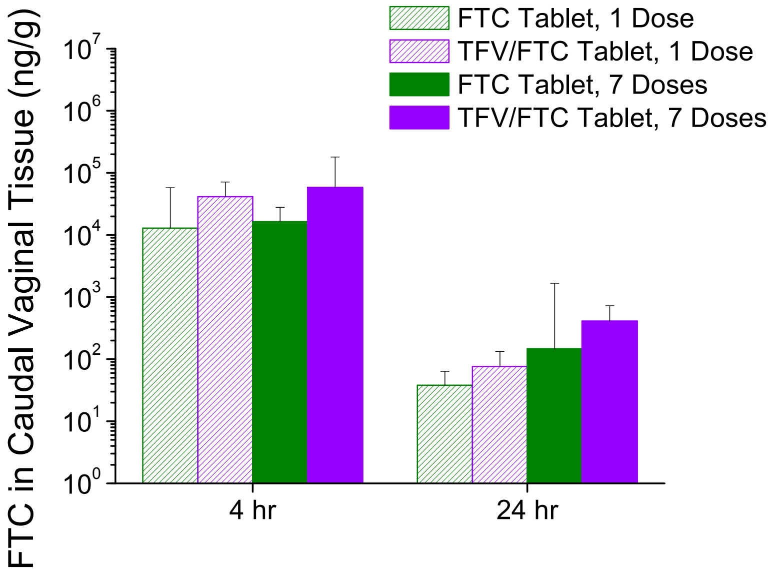

Figure S5. TFV concentrations in iliac lymph nodal tissues at 4 and 24 h following a single or 7 daily doses of intact TFV or TFV/FTC tablets. Data are median + SD (n = 6).

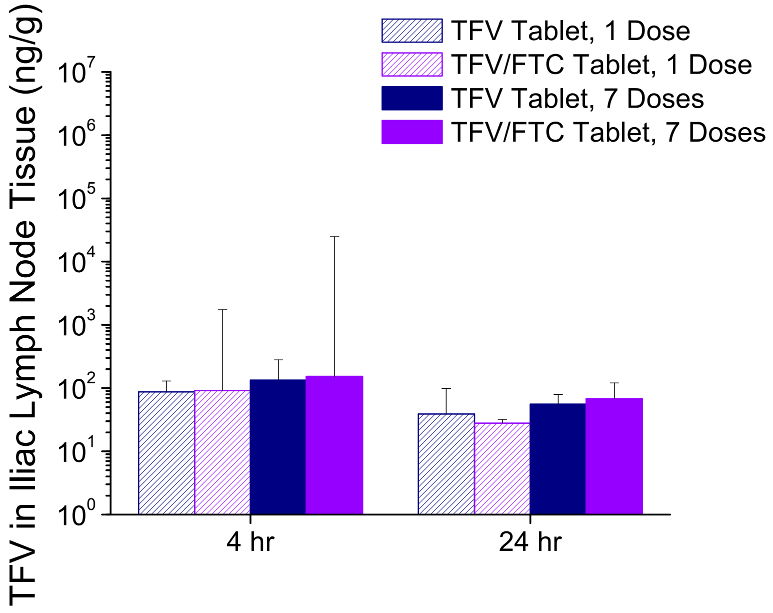

© 2014 by the authors; licensee MDPI, Basel, Switzerland. This article is an open access article distributed under the terms and conditions of the Creative Commons Attribution license (http://creativecommons.org/licenses/by/4.0/).
